# Characterization of Sporadic Creutzfeldt-Jakob Disease and History of Neurosurgery to Identify Potential Iatrogenic Cases

**DOI:** 10.3201/eid2606.181969

**Published:** 2020-06

**Authors:** Tsuyoshi Hamaguchi, Kenji Sakai, Atsushi Kobayashi, Tetsuyuki Kitamoto, Ryusuke Ae, Yosikazu Nakamura, Nobuo Sanjo, Kimihito Arai, Mizuho Koide, Fumiaki Katada, Masafumi Harada, Hiroyuki Murai, Shigeo Murayama, Tadashi Tsukamoto, Hidehiro Mizusawa, Masahito Yamada

**Affiliations:** Kanazawa University Graduate School of Medical Sciences, Kanazawa, Japan (T. Hamaguchi, K. Sakai, M. Yamada);; Hokkaido University, Sapporo, Japan (A. Kobayashi);; Tohoku University Graduate School of Medicine, Sendai, Japan (T. Kitamoto);; Jichi Medical University, Shimotsuke, Japan (R. Ae, Y. Nakamura);; Tokyo Medical and Dental University, Tokyo, Japan (N. Sanjo);; National Hospital Organization Chiba-East Hospital, Chiba, Japan (K. Arai, M. Koide);; Kameda Medical Center, Chiba (F. Katada);; The University of Tokushima, Tokushima, Japan (M. Harada);; International University of Health and Welfare, Narita, Japan (H. Murai);; Tokyo Metropolitan Institute of Gerontology, Tokyo (S. Murayama);; National Center of Neurology and Psychiatry, Kodaira, Japan (T. Tsukamoto, H. Mizusawa)

**Keywords:** Creutzfeldt-Jakob disease, iatrogenic transmission, neurosurgery, prions,, diffusion-weight magnetic resonance imaging, thalamus

## Abstract

We previously reported a phenotype of Creutzfeldt-Jakob disease (CJD), CJD-MMiK, that could help identify iatrogenic CJD. To find cases mimicking CJD-MMiK, we investigated clinical features and pathology of 1,155 patients with diagnosed sporadic CJD or unclassified CJD with and without history of neurosurgery. Patients with history of neurosurgery more frequently had an absence of periodic sharp-wave complexes on electroencephalogram than patients without a history of neurosurgery. Among 27 patients with history of neurosurgery, 5 had no periodic sharp-wave complexes on electroencephalogram. We confirmed 1 case of CJD-MMiK and suspected another. Both had methionine homozygosity at codon 129 of the prion protein gene and hyperintensity lesions in the thalamus on magnetic resonance images of the brain, which might be a clinical marker of CJD-MMiK. A subgroup with a history of neurosurgery and clinical features mimicking dura mater graft-associated CJD might have been infected during neurosurgery and had symptoms develop after many years.

Prion diseases are fatal, transmissible neurodegenerative disorders characterized by deposition of abnormal prion protein (PrP^Sc^) in the brain (*1*). Human prion diseases, which include Creutzfeldt-Jakob disease (CJD), are classified into sporadic, genetic, and acquired forms. Acquired forms can be transmitted from humans to humans, as in cases of iatrogenic CJD or kuru, or from animals or humans to humans, as in cases of variant CJD (vCJD). Thus far, >400 cases of iatrogenic CJD have been reported (*2*). Two major medical procedures, intramuscular injection of cadaveric human growth hormone and cadaveric human dura mater grafting, have caused iatrogenic transmission of PrP^Sc^, and 154 patients, >60% of cases worldwide of dura mater graft–associated CJD (dCJD), have been identified in Japan (*2*,*3*). We previously showed that dCJD could be classified into 2 types, nonplaque and plaque, according to the pathology findings (*4*,*5*). Nonplaque-type dCJD shows clinical features identical to those of typical sporadic CJD (sCJD), but plaque-type dCJD is characterized by atypical pathology and clinical features, including slow disease progression, lack of or late occurrence of periodic sharp-wave complexes (PSWCs) on patients’ electroencephalograms (EEGs), and plaque formation in the brain (*4*,*5*).

Molecular features of plaque type dCJD include methionine (M) homozygosity at codon 129 (129MM) of the prion protein gene (*PRNP*) and a distinctive type of PrP^Sc^ with intermediate electrophoretic mobility (−20 kDa) between type 1 and type 2, which has been designated as intermediate type (type i PrP^Sc^) (*6*,*7*). Clinicopathology studies of patients with dCJD and experimental transmission studies strongly suggest that the combination of 129MM, type i PrP^Sc^, and kuru plaques (CJD-MMiK) is a distinctive hallmark of acquired CJD caused by an infection with the V2 sCJD strain to persons with 129MM (*6*,*8**–**10*). We proposed neuropathology and biochemical criteria to identify CJD-MMiK (*8*,*9*). Surprisingly, we discovered 2 cases of diagnosed sCJD that showed clinical, pathologic, and molecular features identical to CJD-MMiK, which might have been iatrogenically transmitted because 1 of the patients had a history of neurosurgery and the other patient was a neurosurgeon (*8*). These results suggested that CJD-MMiK could be a clue to identify acquired CJD cases among patients with sCJD diagnoses. To identify iatrogenic cases, we investigated clinical features and pathology of patients with diagnosed sCJD and a history of neurosurgery to find features similar to CJD-MMiK.

## Methods

### The Patients

We analyzed patients with suspected prion diseases who were registered by the Creutzfeldt-Jakob Disease Surveillance Committee in Japan during April 1999–February 2016. The surveillance system started in April 1999 and prospectively investigated each patient with a surveillance protocol as previously reported (*5*,*11*,*12*). After obtaining written consent from patients or their families, members of the surveillance committee directly examined the patient and collected data from clinical and pathology records. The surveillance committee reviewed each patient’s case files and assessed EEGs, diffusion-weighted magnetic resonance imaging (DW-MRI), ELISA of cerebrospinal fluid (CSF), and history of neurosurgery and then discussed each case to determine which patients had prion disease. The study protocol was approved by the medical ethics committees of Kanazawa University, Tokyo Medical and Dental University, and National Center of Neurology and Psychiatry in Japan.

For patients with a history of neurosurgery, we collected information about the underlying disease, including the date and hospital in which neurosurgery was performed. We determined which patients had undergone a dura mater graft by reviewing surgical records, querying the neurosurgeon, or examining autopsy findings. 

Members of the committee assessed EEGs and determined whether PSWCs were typical of prion disease or suggested prion disease. Typical cases were deemed positive for prion disease, and the full committee reviewed and discussed suggested cases to decide whether PSWCs were positive or negative for prion disease. 

We examined the cerebral cortices, basal ganglia, and thalamus for hyperintensity lesions on DW-MRI. Committee members defined hyperintensities or submitted scans to the committee’s neuroradiology expert for assessment. For ELISA, we examined the value of 14-3-3 protein in CSF, as previously reported (*13*,*14*). We analyzed *PRNP* for M or valine (V) polymorphism in at codon 129 by using the open reading frame after extracting DNA from patient’s blood, as described earlier (*15*). 

We included definite and probable cases of prion disease in this study and classified them into 4 categories: sporadic, acquired, genetic, and unclassified. We used World Health Organization criteria to diagnose sCJD and iatrogenic CJD (*16*). We diagnosed dCJD when we confirmed dura mater grafting, and considered cases as unclassified CJD when insufficient information on dura mater grafting were available. When we confirmed that dura mater graft was not performed during neurosurgery, we diagnosed sCJD even if the patient had history of neurosurgery not related to dura mater. We classified the patients with pathologically confirmed diagnosis of sCJD and unclassified CJD into 9 categories according to *PRNP* polymorphisms, the type of PrP^Sc^, and pathology seen at autopsy. The 9 subtypes were MM1, MM2-cortical, MM2-thalmic, MM2+1 (pathological features of MM2 and MM1 types of sCJD), MV1, MV2, VV1, VV2, and MMiK. We included patients with definite sCJD and those with probable sCJD and genetic results for *PRNP*, which can distinguish genetic from sporadic prion diseases.

### Clinical and Pathology Findings

We investigated 1,161 cases of sCJD in which no dura mater grafting or other iatrogenic causes were proven. We also investigated 3 patients with unclassified CJD and compared them to patients with and without history of neurosurgery. Among the 1,164 patients, 36 had a history of neurosurgery, but we excluded 9 because they underwent neurosurgeries <1 year before or after the onset of CJD. Our study included 27 patients with history of neurosurgery and 1,128 without history of neurosurgery.

### Statistical Analyses

We used the Student *t* test to compare patients with and without history of neurosurgery, age at onset of CJD, and duration between the onset of CJD and the appearance of akinetic mutism or death. We used the Fisher exact test to analyze sex distribution, negative rate of PSWCs on EEG, and positive rates of 14-3-3 protein and total tau protein (cutoff 1,200 pg/mL) in CSF. We used the χ^2^ test to analyze distribution of M and V polymorphisms. We considered p<0.05 statistically significant. We performed statistical analyses by using SPSS Statistics 22 (IBM, https://www.ibm.com).

## Results

We evaluated clinical features of the patients with and without history of neurosurgery (Table 1). Among the 27 patients with a history of neurosurgery, 5 (18.5%) had no PSWCs on EEG, but only 64/1,121 (5.7%) patients without history of neurosurgery demonstrated absence of PSWCs on EEG. However, we did not identify statistically significant differences for age at onset of CJD, sex, distribution of polymorphisms, disease duration, or positive rate of 14-3-3 protein or total tau protein in CSF.

**Table 1:** Comparison of the clinical features of patients with Creutzfeldt-Jakob disease with and without history of neurosurgery*

Patient characteristics	Neurosurgery	No neurosurgery	p value
Total no. patients	27	1,128	
Sex, no. (%)			
F	17 (63.0)	647 (57.4)	0.353
M	10 (37)	481 (42.6)	
Age at onset, y + SD (range)	71.0 + 8.8 (49–88)	68.7 + 9.6 (30–91)	0.217
Disease duration, mo + SD (range)†	6.1 + 7.8 (1–28)	6.7 + 12.0 (0–171)	0.823
Duration from neurosurgery to onset of CJD, y + SD (range)	15.0 + 9.1 (1–35)		
Polymorphism at codon 129 of prion protein gene, no. (%)			
MM	25 (92.6)	1,101 (97.6)	0.138
MV	2 (7.4)	22 (1.9)	
VV	0	5 (0.4)	
Negative rate of PSWCs on EEG, no. (%)	5 (18.5)	64/1,121 (5.7)	0.019
14-3-3 protein in CSF, no. tested/no. positive (%)	20/22 (90.9)	675/803 (84.1)	0.300
Tau protein in CSF, cutoff 1,200 pg/mL, no. tested/no. positive (%)	13/14 (92.9)	503/567 (88.7)	0.523

*CJD, Creutzfeldt-Jakob disease; CSF, cerebrospinal fluid; EEG, electroencephalogram; PSWCs, periodic sharp-wave complexes †Disease duration of CJD: duration between the onset of CJD and the appearance of the akinetic mutism or death in the patients who died without akinetic mutism

Among the 27 sCJD patients with history of neurosurgery, 5 (18.5%) had atypical CJD features, including no PSWCs on EEG during their illnesses (Table 2). Among the 5 patients with atypical sCJD, the median age (+ SD) at CJD onset was 65.4 (+ 9.6) years (range 49–75 years); 4 were women and 1 was a man. Neurosurgery occurred during 1977–1993. The average time between neurosurgery and onset of CJD was 18.0 (+ 8.8) years (9–30 years), and the average time between onset of CJD and the appearance of the akinetic mutism or death was 17.0 (+ 7.7) months (6–28 months). Among the 5 patients with atypical sCJD, 2 patients were unclassified because we had no or insufficient information on dura mater graft during neurosurgery. The other 3 patients were determined to have sCJD without a history of dura mater–related neurosurgery. None of the 5 patients with atypical CJD had *PRNP* mutation, but all had 129MM.

**Table 2:** Patients with history of neurosurgery who had no periodic sharp-wave complexes on electroencephalogram during course of Creutzfeldt-Jakob disease*

Patient no.	Age at CJD onset, y/sex	Year of neurosurgery; reason	Time from neurosurgery to onset of CJD, y	Initial symptoms	Disease duration, mo†	Codon 129 of *PRNP*	Lesions on DW-MRI	Pathology findings
1	75/M	1977; head trauma	30	Dementia	11	MM	CC, BG	ND
2	49/F	1985; subarachnoid hemorrhage	9	Insomnia	28	MM	ND	MM2-T
3	75/F	1985; tumor	14	Drowsiness, gait disturbance	6	MM	BG, Th	CJD-MMiK
4	63/F	1985; tumor	27	Gait disturbance	19	MM	BG, Th	ND
5	64/F	1993; subdural hematoma	10	Visual impairment	21	MM	CC	MM2-C

*Among the patients with history of neurosurgery, average time (+ SD) from neurosurgery to onset of CJD was 18.0 (+ 9.8) years and average age at neurosurgery was 47.2 (+ 10.2) years. However, among 22 patients with PSWCs on EEG, average time (+ SD) from neurosurgery to onset of CJD was 14.3 (± 9.1) years and average age at neurosurgery was 58.0 (± 12.2) years. We noted no statistically significant differences between patients with and without PSWCs on EEG in relation to time between neurosurgery and onset of CJD or in age at neurosurgery. BG, basal ganglia; CC, cerebral cortex; CJD, Creutzfeldt-Jakob disease; DW-MRI, diffusion weighted images on magnetic resonance imaging; MM, methionine homozygous; MM2-C, MM2-cortical type sporadic CJD; MM2-T, MM2-thalamic type sporadic CJD; ND, not done; *PRNP*, prion protein gene; Th, thalamus. †Disease duration is the time between onset of CJD and the appearance of akinetic mutism or death.

DW-MRI was performed in 4/5 atypical patients and we noted hyperintensity lesions in various brain regions (Table 2). In patient 3 (Appendix Figure 1, panel A) and patient 4 (Appendix Figure 1, panel B), images showed hyperintensity lesions in bilateral thalamus and bilateral basal ganglia.

Autopsies were performed on 3/5 patients with atypical CJD (patients 2, 3, and 5); all cases are reported in detail elsewhere (*8**,**17**–**19*). We assessed the subtypes of CJD with pathology and biochemical analysis of the brain and noted MM2-thalamic type in patient 2, CJD-MMiK in patient 3, and MM2-cortical type sCJD in patient 5 (Table 2). Patient 3 did not receive a dura mater graft according to neurosurgery records, and none was observed at autopsy.

Among patients who had not undergone neurosurgery, 64/1,121 showed no PSWCs on EEG, including 51 (79.7%) with 129MM, 4 (6.3%) with 129VV, and 9 (14.1%) with 129MV. Western blot of brain tissue performed on 25 patients revealed 5 with MM1 type CJD, 9 with MM2 type, 5 with MM2+1 type, 4 with MV2 type, and 2 with VV2 type. DW-MRI was available for 55/64 patients without PSWCs on EEG, 44 patients with 129MM, 8 with 129MV, and 3 with 129VV. On DW-MRI, 10/55 patients had hyperintensity lesions in the thalamus (Table 3), 3 of whom had 129MM, including 2 patients with MM2+1 type. Slight thalamic signal increase was observed in mediodorsal nuclei in all 3 patients with 129MM (Appendix Figure 2).

**Table 3:** Patients without history of neurosurgery who had no periodic sharp-wave complexes during duration of CJD and hyperintensity lesions in thalamus diffusion-weighted magnetic resonance imaging of the brain*

Patient no.	Age at CJD onset, y/sex	Codon 129 of *PRNP*	Hyperintensity lesions on DW-MRI	Pathology findings
1	58/F	MM	CC, BG, Th	MM2+1
2	65/M	MM	CC, Th	MM2+1
3	61/F	MM	CC, BG, Th	ND
4	58/M	MV	BG, Th	MV2
5	73/F	MV	CC, Th	MV2
6	65/F	MV	CC, BG, Th	ND
7	65/F	MV	CC, Th	ND
8	75/F	VV	BG, Th	VV2
9	69/M	VV	BG, Th	VV2
10	52/M	VV	CC, BG, Th	ND
*BG, basal ganglia; CC, cerebral cortex; CJD, Creutzfeldt-Jakob disease; DW-MRI, diffusion-weighted magnetic resonance imaging; MM, methionine homozygous; MM2+1, pathological features of MM2 and MM1 types of sporadic CJD; MV, methionine-valine; Th, thalamus; VV, valine homozygous.				

## Discussion

We identified 27/1,155 (2.3%) patients with diagnosed sCJD or unclassified CJD who had neurosurgery >1 year before the onset of CJD. We also noted that more patients with history of neurosurgery had an absence of PSWCs on EEG than patients who had not had neurosurgery. Furthermore, 5/27 patients with history of neurosurgery had atypical clinical features, including no PSWCs on EEG for the duration of CJD. Of note, among 5 patients with 129MM, 2 had hyperintensity lesions in the thalamus on DW-MRI, 1 of whom was confirmed to have CJD-MMiK by autopsy.

In our previous study, patients with acquired CJD-MMiK showed no PSWCs <1 year after onset of CJD (*4*,*5*). Among 3 autopsies for cases without PSWCs on EEG and a history of neurosurgery, 2 patients were determined to have definite MM2 type sCJD with atypical clinical features (Table 2), as previously reported (*19**–**22*). One had MM2-cortical form and the other had MM2-thalamic form. Our study proved 1 patient had CJD-MMiK (Table 2), which we reported previously (*8*,*17*). Among the patients with history of neurosurgery, no PSWCs on EEG might suggest possibility of acquired CJD-MMiK.

The patient determined to have CJD-MMiK showed hyperintensity lesions in bilateral thalamus in addition to bilateral basal ganglia (Appendix Figure 1, panel A). Several patients with plaque type dCJD showed hyperintensity lesions in thalamus on DW-MRI or proton density-weighted MRI (*5**,**23**–**25*; K. Sakai et al., unpublished data). A previous study on MRI of sCJD showed that the absence of thalamic signal increase differentiated MM1 from other types of sCJD, but thalamic involvement was occasionally observed in MM2-cortical type sCJD (*26*). In our study, thalamic signal increase was observed in 3/44 patients with diagnosed sCJD, and 129MM patients without history of neurosurgery showed only slight signal increases in mediodorsal nuclei. These results suggest that hyperintensity lesions in the thalamus on DW-MRI in patients with 129MM might be a clinical marker of CJD-MMiK, but we cannot exclude the possibility of MM2-cortical type sCJD. Further MRI studies of plaque type dCJD cases with CJD-MMiK is ongoing.

According to a previous study on MRI of sCJD, the most characteristic MRI lesion patterns in the patients with MV2 and VV2 type sCJD, who were infected with V2 sCJD strain, shows predominant involvement of thalamus and basal ganglia (*26*). Because transmission of V2 sCJD strain to persons with 129MM causes CJD-MMiK (*7*–*10*), thalamic involvement frequently was observed, as we noted in the case we identified in this study. 

One of the 2 atypical patients with history of neurosurgery, patient 4 (Table 2), did not have an autopsy but had signs and symptoms of CJD-MMiK. Patient 4 had bilateral hyperintensity lesions in thalamus on DW-MRI (Appendix Figure 1, panel B), no PSWCs on EEG, methionine homozygosity at codon 129 of *PRNP*, gait disturbance as an initial symptom, and long disease duration (19 months), similar to the features of the patients with plaque-type dCJD, suggesting that this patient had CJD-MMiK. However, no information about dura mater grafting at the neurosurgery is available for this patient.

We identified 1 patient with definite CJD-MMiK and another patient with suspected CJD-MMiK among 1,155 patients with diagnosed sCJD or unclassified CJD, suggesting that these 2 patients could have iatrogenic CJD. Several epidemiologic studies have shown no association between surgery and the onset of sCJD, but others show notable association when special conditions are met, such as lag periods >20 years between surgery and CJD onset and when patients are <30 years of age at the time of surgery (*27*,*28*). For the 2 patients with confirmed and suspected CJD-MMiK in our study, patient 3 underwent neurosurgery at 61 years of age, 14 years before CJD onset. Patient 4 underwent neurosurgery at 36 years of age, 27 years before CJD onset. Both of these patients’ illness onset fall into the range noted between surgical procedure to illness onset (1–30 years) among 154 patients with dCJD in Japan (*29*). Compared with other surgical procedures, neurosurgery followed by protracted survival is remarkably infrequent and therefore is difficult to study. Our results showed that few patients mimic the findings of MMiK type CJD, which might provide clues supporting the presence of a subgroup of CJD transmitted by neurosurgery after a long time lag without evidence of reuse of contaminated instruments on a CJD patient. An experimental research study provides strong support for an eventual transmission of CJD by general surgery in addition to neurosurgery (*30*), and high-quality epidemiologic research on neurosurgical risk for CJD is needed. Furthermore, we cannot find iatrogenic CJD patients who transmit M1 sCJD strain to persons with 129MM because the transmission would result in MM1 type neuropathology. A new method to detect transmission of M1 sCJD strain to persons with 129MM is needed.

Thus far, a patient with cadaveric pituitary-derived human growth hormone (hGH)–associated CJD in the United Kingdom was reported to have 129MM, type i PrP^Sc^, and kuru plaques (*31*), suggesting that patients without history of neurosurgery could develop CJD-MMiK. In that case, the patient received hGH through intramuscular injection. Another 2 patients with hGH-associated CJD were reported to have 129MM and type i PrP^Sc^ (*32*,*33*). Unfortunately, detailed clinical information, including brain MRI, is not available for these patients, and we cannot compare the clinical manifestations with those for plaque type dCJD in Japan. Further studies for CJD-MMiK in patients with hGH-associated CJD are needed.

In conclusion, among 1,155 patients with diagnosed sCJD or unclassified type CJD, 2 patients with a history of neurosurgery had atypical clinical and neuropathologic features similar to acquired CJD-MMiK. A subgroup of sCJD mimicking features described in dCJD might have been transmitted by neurosurgery after long time lag. Hyperintensity lesions in the thalamus on DW-MRI in patients with 129MM might be a clinical marker of acquired CJD-MMiK. For epidemiologic surveillance studies, we propose clinical diagnostic criteria of sCJD potentially transmitted by neurosurgical procedures, which includes the criteria of possible, probable, or definite sCJD; methionine homozygosity at codon 129 of *PRNP*; no PSWCs on EEG; and hyperintensity lesions in the thalamus on DW-MRI.

Appendix Additional information on characterization of sporadic Cruetzfeld-Jakob disease and history of neurosurgery to identify potential iatrogenic cases.
