# Supplementary material for: Characterization of Sporadic Creutzfeldt-Jakob Disease and History of Neurosurgery to Identify Potential Iatrogenic Cases
Source: Emerg Infect Dis. 2020 Jun;26(6):1140–6. doi: 10.3201/eid2606.181969 (PMC7258447; doi:10.3201/eid2606.181969)
Supplement: Appendix — Additional information on characterization of sporadic Cruetzfeld-Jakob disease and history of neurosurgery to identify potential iatrogenic cases. [file 18-1969-Techapp-s1.pdf]

Article DOI: https://doi.org/10.3201/eid2606.181969

Characterization of Sporadic Creutzfeldt-Jakob Disease and History of Neurosurgery to Identify Potential Iatrogenic Cases

Appendix

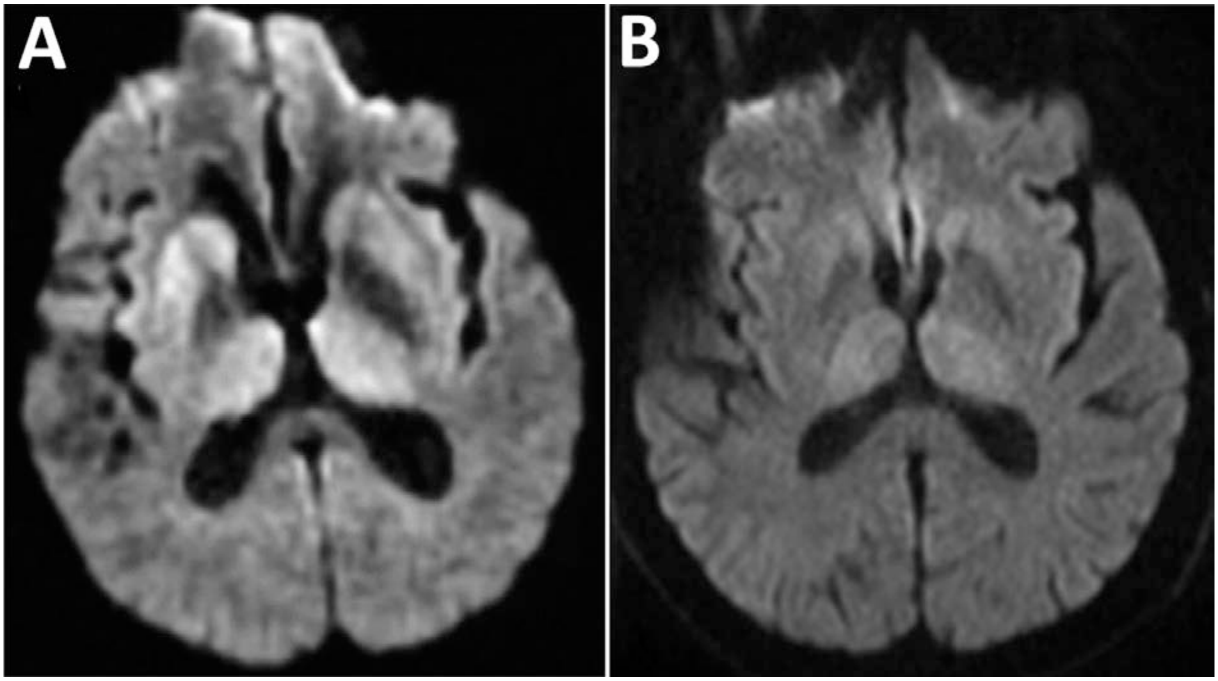

Appendix Figure 1. Diffusion-weighted magnetic resonance imaging (DW-MRI) of the brain in patients with history of neurosurgery who had no periodic sharp-wave complexes during duration of Creutzfeldt-Jakob disease (CJD). A) DW-MRI of patient 3 (Table 2) at 8 months after the onset of CJD (*27*); an autopsy examination determined that the patient had acquired CJD-MMiK. Scan shows hyperintensity lesions in bilateral thalamus in addition to basal ganglia lesions. B) DW-MRI of patient 4 (Table 2) at 9 months after the onset of CJD showing hyperintensity lesions in bilateral thalamus and bilateral basal ganglia.

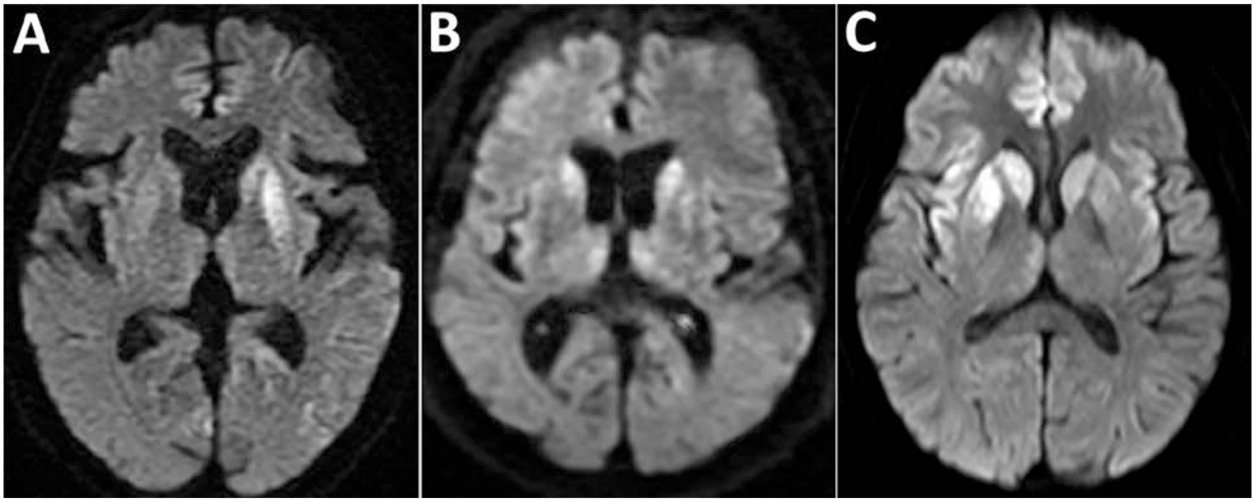

Appendix Figure 2. Diffusion-weighted magnetic resonance images (DW-MRI) of the brain in patients without history of neurosurgery who had no periodic sharp-wave complexes during duration of Creutzfeldt-Jakob disease (CJD), methionine homozygote at codon 129 of prion protein gene, and increasing signals in thalamus. A) DW-MRI of patient 1 (Table 3), who was determined to have MM2 and MM1 type sporadic Creutzfeldt-Jakob disease (sCJD), showing slightly increased signals in bilateral mediodorsal nuclei of thalamus, bilateral cerebral cortices, and left basal ganglia. B) DW-MRI of patient 2 (Table 3), who was determined to have MM2-cortical and MM1 type sCJD, showing slightly increased signals in bilateral mediodorsal nuclei of thalamus, cerebral cortices, and bilateral basal ganglia. C) DW-MRI of patient 3 (Table 3) showing slightly increased signals in bilateral mediodorsal nuclei of thalamus, cerebral cortices, and bilateral basal ganglia.
